# Supplementary material for: Clarifying terminology and definitions in education services for mental health users: A disambiguation study
Source: PLoS One. 2024 Jul 3;19(7):e0306539. doi: 10.1371/journal.pone.0306539 (PMC11221696; doi:10.1371/journal.pone.0306539)
Supplement: S1 Table — (DOCX) [file pone.0306539.s001.docx]

## Supporting Information

## Table 1. Questions of the expert interview

| Aim of the meeting is to see whether the information we have gathered so far is correct and whether DESDE-LTC is able to classify the services in the education sector accurately | | | | |
| --- | --- | --- | --- | --- |
| How can the education sector be defined? |  |  |  |  |
|  |  |  |  |  |
| **General questions** | | |  |  |
| Are the experts aware of the problem? Is coding of services is an issue? Is there an official listing of education services and interventions and their related costs? | | | | |
| Are the experts aware of any cost-effectiveness analyses in the country or in the region? | | | | |
| Do the experts have access to lists of key planners in the sector? | | |  |  |
| Do the codes reflect the characteristics of the education system? | | |  |  |
| Is it important to incorporate the distinction into primary and secondary education services? | | | | |
| Which ministry or official organization is responsible for the education sector? | | | | |
| What are the key professionals with university degree in education (equivalent to physician (to differentiate the type of residential care), nurse, psychologist etc.)? | | | | |
|  |  |  |  |  |
| **Specific questions on the general services** | | | | |
| Item #1 (special education services): Check definition with experts, whether type of care will depend on the specific needs | | | | |
| Item #41 (special education boarding school): Ask if these are meso-organizations; the same target group and the same administration. Center with different codes, entities | | | | |
| Item #43 (Higher education school (university, college, vocational school)): Can the population be both underage and adult? | | | | |
| Are there differences in a boarding school with 24 h teachers and other that don't? is that a possibility? Who takes care in this case? | | | | |
| Are there professionals available 'on call' when they are needed? | | |  |  |
