# Supplementary material for: Clarifying terminology and definitions in education services for mental health users: A disambiguation study
Source: PLoS One. 2024 Jul 3;19(7):e0306539. doi: 10.1371/journal.pone.0306539 (PMC11221696; doi:10.1371/journal.pone.0306539)
Supplement: S2 Table — (DOCX) [file pone.0306539.s002.docx]

## Table 2. Commensurability assessment (Unit of analysis)

| **#** | **Item** | **Service description** | **Rating level 1** Accurate = service Vague = could be a service, but not clear Confusing = definitely not a service | **Clarification** |
| --- | --- | --- | --- | --- |
| 1 | Special education services | Special education for childern who are not able to be supported in regular school classes concerning personal developments and achievements | Accurate |  |
| 2 | Learning therapy | Includes e.g. the diagnosis of learning disability with regards to reading, writing, calculating or weak concentration; individual therapy and treatment plans; counselling and thereby fostering individual strenghts, skills and talents | Confusing | Therapy is an intervention, not a service |
| 3 | Home education | Schooling provided by the children's parents in the home environment | Confusing | Not a service provided by professional team, intervention or procedure |
| 4 | School-based health promotion interventions | Programs/strategies organized in the school environment aimed at producing behavior changes or improving health status of the students | Confusing | Interventions |
| 5 | Liason teacher | Teacher who is responsible for to aid and assist when difficulties occur amongst students or between student and teacher | Confusing | Professional |
| 6 | Compensation for disadvantages | Compensation for disadvantages such as learning disabilities, difficulties regarding concentration, etc. in for example, giving a student more time to complete a task | Confusing | Consequence |
| 7 | Special needs diagnostics | Form of diagnostics to detect potential need for special needs education | Confusing | Procedure, not a service |
| 8 | Counseling of legal guardians | Counseling of legal guardians concerning development, support and abilities of their child | Confusing | Procedure, not a service |
| 9 | Student counselling | After-school counselling for students who experience difficulties with school performance | Vague | Not clear what is understood by counselling |
| 10 | Temporary study group | Study group for children with severe social and learning disabilities | Confusing | Intervention, not a service |
| 11 | Social and educational therapy boarding school | Tutoring of students in residential groups, special boarding schools, etc. with the help of therapists, social workers, teachers and other professionals | Confusing | Procedure, not a service |
| 12 | Night school | Adult learning school that holds classes in the evening or at night to accommodate people who work during the day | Accurate |  |
| 13 | Attendance officer | School official who deals with the issues of absenteeism among students | Confusing | Professional |
| 14 | Student transport to special education facility | Services aimed at transporting students to special education facility | Accurate |  |
| 15 | Student-related financing | Financial support for schools meant for students who experience learning disabilities, e.g. additional guidance and adapted lesson materials | Confusing | Financing is not a service |
| 16 | Change in school readiness | Due to mental illness, child enters school less ready to engage in and benefit from early learning experiences that best promote the child’s success | Confusing | Consequence |
| 17 | Problems with school entry | Delayed school entry due to child's mental illness | Confusing | Consequence |
| 18 | Learning disabilities | Impaired learning developing e.g. related to reading, spelling and/or calculating | Confusing | Consequence |
| 19 | Cognitive deficits | Deficits in overall intelligence | Confusing | Consequence |
| 20 | Low school adaptation/competence | Impaired adaptation to the school environment | Confusing | Consequence |
| 21 | Low school participation/engagement | Lower involvement in school activities | Confusing | Consequence |
| 22 | Low school attainment/productivity/performance | Student's absenteeism/presenteeism due to morbidity or due to death of a sick relative | Confusing | Consequence |
| 23 | Grade retention | Repetition of a school year in case a student is not entitled to advance to the next year | Confusing | Consequence |
| 24 | Disrupted school experience | Interrupted/disturbed learning experience | Confusing | Consequence |
| 25 | Teacher-student conflicts | Conflicts that arise between a teacher and a student | Confusing | Consequence |
| 26 | School droupout/pre-mature leave | Pre-mature leave from school without completing educational level/degree | Confusing | Consequence |
| 27 | Indirect effect of premature school leave/drop-out | Negative effects of pre-mature school leave that appear during the life course of a student e.g. less job opportunities, lower socio-economic status, worse health | Confusing | Consequence |
| 28 | (Social) reintegration | Measures taken by the school to promote integration after a childs time of abscence due to illness or hospital stay | Confusing | Consequence |
| 29 | Inclusion | Enabling individual learning for all students regardless of (social) disabilites | Confusing | Consequence |
| 30 | Refusal of admission | The admission authority of education facility refused to admit a student | Confusing | Consequence |
| 31 | Change in educational level | Negative effect of mental illness on school performance leading to change in the educational level towards lower level | Confusing | Consequence |
| 32 | Exemption from compulsory education | Student is relieved from the obligation to attend school | Confusing | Consequence |
| 33 | Talent development | Impaired development of student's skills and competencies | Confusing | Consequence |
| 34 | Discrimination | Students being treated less well or put at a disadvantage for a reason that relates to their disability | Confusing | Consequence |
| 35 | Peer relations | Relationship between students, e.g. conflict, bullying | Confusing | Consequence |
| 36 | Suspension | Mandatory leave assigned to a student as a form of punishment | Confusing | Consequence |
| 37 | Negative feelings about school | Student's dislike of school | Confusing | Consequence |
| 38 | Classroom behaviour | Student's behavior during lessons | Confusing | Consequence |
| 39 | Special education day school | A day school providing education and guidance for pupils with special educational needs | Accurate |  |
| 40 | Day school | A day school providing regular education (language, math, history) to groups of children | Accurate |  |
| 41 | Special education boarding school | Facility providing care, support, education and employment to people of all ages with severe (intellectual) disabilities and complex behavioral and / or psychiatric problems. This facility offers special education during the day for groups of children and young people (max 16) who experience a structural limitation in their educational participation due to their behavioral disabilities or psychiatric problems | Accurate |  |
| 42 | Boarding school | A small scale school offering regular education during the day as well as flexible boarding and full boarding facilities | Accurate |  |
| 43 | Higher education school (university, college, vocational school) | A high-level educational institution offering upper-secondary or tertiary education services to groups of students | Accurate |  |
| 44 | Education support at your place of study (e.g. tutoring, additional lessons) | Additional educational support provided as an outpatient service at a regular primary/secondary school to students with learning difficulties (often referred to as remedial teaching) | Accurate |  |
| 45 | Education support in a private setting (e.g. private tutoring) | Additional educational support provided as an outpatient service in a private setting to students with learning difficulties (often referred to as remedial teaching) | Accurate |  |
| 46 | Dormitory (boarding school, college) | A university or college hall of residence or hostel | Confusing | Facility, not a service |
