# Supplementary material for: Clarifying terminology and definitions in education services for mental health users: A disambiguation study
Source: PLoS One. 2024 Jul 3;19(7):e0306539. doi: 10.1371/journal.pone.0306539 (PMC11221696; doi:10.1371/journal.pone.0306539)
Supplement: S3 Table — (DOCX) [file pone.0306539.s003.docx]

## Table 3. Spanish services and programmes

| **County** | **Name ENG** | **Description ENG** | **Disease area** | **Type of data source** | **Source** | DESDE-LTC code | Expert comments |
| --- | --- | --- | --- | --- | --- | --- | --- |
| AACC - ANDALUSIA | Centers for behavioral disorders | Internment centers different from those envisaged in the penitentiary legislation for the execution of criminal convictions and precautionary measures deprived of liberty imposed on those of legal age, whose purpose is to comply with the judicial measures involving deprivation of liberty from a double perspective, sanctioning and educational, configuring a comprehensive care framework and a highly structured educational intervention oriented towards personal and social development, in accordance with the principles of equality between women and men, in order to facilitate the evolution of the minor offender to enter life on freedom. The centers that execute therapeutic internment measures have an autonomous unit for this purpose. The center's educational project specifically contemplates the program of action of the therapeutic unit, taking into account all its unique characteristics, while promoting the participation of minors subject to measures of therapeutic internment in the general activities of the center. The therapeutic units give integral attention to the needs of the minors and are trained to face the needs related to the mental health of the minors as well as the specific ones derived from the abusive consumption of addictive substances. | all | regional report | La Salud Mental en Andalucía, 2008-2013 |  | Justice. Consider here juvenile justice educational centres . SE- Ca D4.2gj |
| AACC - ANDALUSIA | Special education specific centers | Internment centers different from those envisaged in the penitentiary legislation for the execution of criminal convictions and precautionary measures deprived of liberty imposed on those of legal age, whose purpose is to comply with the judicial measures involving deprivation of liberty from a double perspective, sanctioning and educational, configuring a comprehensive care framework and a highly structured educational intervention oriented towards personal and social development, in accordance with the principles of equality between women and men, in order to facilitate the evolution of the minor offender to enter life on freedom. The centers that execute therapeutic internment measures have an autonomous unit for this purpose. The center's educational project specifically contemplates the program of action of the therapeutic unit, taking into account all its unique characteristics, while promoting the participation of minors subject to measures of therapeutic internment in the general activities of the center. The therapeutic units give integral attention to the needs of the minors and are trained to face the needs related to the mental health of the minors as well as the specific ones derived from the abusive consumption of addictive substances. | all | regional report | La Salud Mental en Andalucía, 2008-2013 | SE-[F7-F8, ICF3] D4.2g | (Type D) |
| AACC - ANDALUSIA | Specialized educational guidance teams | Specialized educational guidance teams | all | regional report | La Salud Mental en Andalucía, 2008-2013 | SE-Cc [F84.0; F9] I1.2.1s | Recently created (15 years ago). They are provincial teams composed of guidance professionals who specialize in a field of special education needs: autism, severe behavioral disorder, physical disability, hearing impairment, visual impairment (has an agreement with the Spainish organization ONCE and the position is determined by them), high abilities and recently, learning difficulties and early care. They serve an entire province and advise for education intervention with this type of students. Professionals access thorugh public calls. Their main function is to make an assessment and a report that supports or not the opinion of the local education guidance teams for autism and severe behavioural problems for a specific center. They are located in the territorial delegation. They are called teams but they are usually single-handed professionals. There are teams that have a teacher and others do not. They were created as a response to complaints to the administration. |
| AACC - ANDALUSIA | Educational Guidance Team | Educational Guidance Team | all | regional report | La Salud Mental en Andalucía, 2008-2013 | SE-Cc [F7-F8] I1.2.1 |  |
| AACC - ANDALUSIA | Departments of Orientative Education | Departments of Orientative Education | all | regional report | La Salud Mental en Andalucía, 2008-2013 | SE-CA- I1.2.1 O8.2 |  |
| AACC - ANDALUSIA | FORMA JOVEN Points | The Forma Joven strategy is developed in the Youth Forma Points, located in the different spaces frequented by the adolescent and youth population, such as secondary schools, workshop schools, universities, leisure spaces, community centers, etc. | all | regional plan | Plan Integral de Salud Mental de Andalucía: Informe de Evaluación Interna del II PISMA (2008‐2013) |  | Not service |
| AACC - ANDALUSIA | Combined schooling |  |  |  | Expert |  | Not a service. The child goes to the special education center for receiving some type of specialised care such as physiotherapy or behavior modification and then he goes to the ordinary center. In practice it is difficult to carry the child from one place to another. |
| AACC - ANDALUSIA | Education in hospitals |  |  |  | Expert | SE- CA [F0-F9; ICD10] D4.2h |  |
| AACC - ANDALUSIA | Teacher education centre |  |  |  |  | SE-AX D4.2g | Training for teachers that is voluntary and free, it is organized by school teachers/educators that behave as counsellors for this specific task. This training represents an important part of the budget for education |
| AACC - BASQUE COUNTRY | Educational therapeutic centers | Attention to serious childhood mental disorder in the educational field | all | regional plan | Estregia en salud mental en la CAPV-ESM 2010 |  | Not available in Andalucía |
| AACC - BASQUE COUNTRY | Juvenile Justice Educational Center | Juvenile Justice Educational Center | all | regional plan | IV PLAN DE JUSTICIA JUVENIL. Tomo I. 2014 – 2018 |  |  |
| AACC - BASQUE COUNTRY | Territorial Center for Hospital, Domestic and Therapeutic-Educational Care | Their purpose is educational and therapeutic support for students who can not attend their school in a normal way by medical prescription, either because they are hospitalized in a health institution, in a home hospitalization or because they are included in an educational therapeutic program. Their functions are detailed in the Decree of creation (DECREE 266/2006, of December 26. BOPV 5-01-2007). | all | regional protocol | MODELO DE ATENCIÓN TEMPRANA PARA LA COMUNIDAD AUTÓNOMA DEL PAÍS VASCO |  |  |
| AACC - BASQUE COUNTRY | Special Education Centers | Special Education Centers | all | regional protocol | MODELO DE ATENCIÓN TEMPRANA PARA LA COMUNIDAD AUTÓNOMA DEL PAÍS VASCO |  |  |
| AACC - BASQUE COUNTRY | Berritzegunes | Configured as support services, they are educational instruments for innovation and improvement of education. Its framework of action will be all the teaching centers of the Autonomous Community of the Basque Country, from both public and private networks. | all | regional protocol | MODELO DE ATENCIÓN TEMPRANA PARA LA COMUNIDAD AUTÓNOMA DEL PAÍS VASCO |  |  |
| AACC - CATALONIA | Center with Educational Resources for Students with Developmental Disorders and Behaviors (CRETDIC) | It is a specific educational support service, advice and training in the regular educational centers of children, primary and secondary sustained with public funds in relation to the problem posed by students with disorders of behavior, autism and mental disorders. | all | regional plan | Estratègia 2017-2019 - Pla integral d’atenció a les persones amb trastorn mental i addiccions |  |  |
| AACC - CATALONIA | Integral services for guidance, counselling and support for labor market placement in the ordinary market (SIOAS) | The purpose of the service is to increase the level of employability and labor insertion and facilitate the social and labor adaptation of disabled workers with special difficulties in labor market insertion in companies in the ordinary labor market. | all | regional plan | Estratègia 2017-2019 - Pla integral d’atenció a les persones amb trastorn mental i addiccions |  |  |
| AACC - CATALONIA | Support Units for students with special educational needs (USEE) | The units of support for students with special educational needs (USEE) are extraordinary endowments of professionals that are incorporated into the school and institute staff as intensive and conjunctural resources to cater for students with special educational needs that need it. | all | regional plan | Estratègia 2017-2019 - Pla integral d’atenció a les persones amb trastorn mental i addiccions |  |  |
| AACC - CATALONIA | Early Childhood and Early Childhood Development Centers (CDIAP) | Coordination between APS pediatric teams, CSMIJs, childhood and early care development centers (CDIAP) and CASs, schools, social services and other community services that contribute to the promotion of neurodevelopment of children. This action must be carried out within the framework of the Comprehensive Plan for mental health and addictions | all | regional plan | Estratègies 2017-2020- Pla director de salut mental i addiccions |  |  |
| AACC - CATALONIA | Territorial functional units of integrated care | Form of coordination between APS pediatric teams, CSMIJs, childhood and early care development centers (CDIAP) and CASs, schools, social services and other community services that contribute to the promotion of neurodevelopment of children. This action must be carried out within the framework of the Comprehensive Plan for mental health and addictions | all | regional plan | Estratègies 2017-2020- Pla director de salut mental i addiccions |  |  |
| AACC - CATALONIA | Residential educational therapeutic unit for children and adolescents in a situation of vulnerability. Center Acompanya'm | The main objective of the Unit Accompany me is to promote the recovery of each teenager or child and to make it easier for them to return to their social and family environment. | all | regional plan | Pla director de salut mentali addiccions |  |  |
| AACC - CATALONIA | Psychopedagogical counseling and counseling teams (EAP). | They are counseling and psychopedagogical counseling teams that support teachers and educational centers in response to the diversity of students and in relation to students who have special educational needs as well as their families. EAPs are part of the educational services area (SEZ) in conjunction with the pedagogical resources centers (CRP) and language, intercultural and social cohesion (ELIC) advice teams. | all | regional plan | Priorització d’actuacions 2014-2016 - Pla integral d’atenció a les persones amb trastorn mental i addiccions |  | Equivalent to Equipos de Orientación Educativa in Andalucía |
| AACC - CATALONIA | Shared schooling units (UEC) | These spaces allow students at risk of social exclusion and with problematic behavior to temporarily school in shared units between an educational center and an external entity that offers specific activities adapted to their needs. The objective of these spaces is that students can continue their academic preparation and reinforce their individual capacities. The UEC allows students to be educated from 14 to 16 years old, or who attended third and fourth grade of ESO. The attendance of the students in the unit supposes a schooling shared with the educational center where they are enrolled and has a temporary nature. There are two types of shared schooling: one full time, with curricular linguistic, social, scientific, technological and practical contents; and another one at part time, which includes learning from the practical field. The entities that are presented to the competition to host the UEC have to prove technical solvency of the equipment and the professionals that will attend the students. They must also provide, among other services, personalized attention to the students that includes a reinforcement in a smaller environment and with a more flexible organization. They will also have to opt for personal guidance through the elaboration of a training itinerary taking into account their age, motivation, interests and personal capacities, in order to encourage greater involvement of the students. | all | regional plan | Priorització d’actuacions 2014-2016 - Pla integral d’atenció a les persones amb trastorn mental i addiccions |  | Also called escolarzación combinada en Andalucía |
