# Supplementary material for: Clarifying terminology and definitions in education services for mental health users: A disambiguation study
Source: PLoS One. 2024 Jul 3;19(7):e0306539. doi: 10.1371/journal.pone.0306539 (PMC11221696; doi:10.1371/journal.pone.0306539)
Supplement: Attachment — Submitted filename: Article report 25.02.2024.pdf [file pone.0306539.s004.pdf]

Dear Editor,

Greetings,

Thank you for your trust.

According to the study “The Role of Educational Games in Developing Social Interaction Skills Among Kindergarten Children from The Point of View of Nannies in The Jerusalem Governorate”.

The title holds significant importance, as do the objectives of the study. However, it is possible that the approach of the study (qualitative and quantitative) encompasses all main parts of the research, commencing with the abstract. It is advisable to refrain from explicitly mentioning the questionnaire in two brackets for the quantitative aspect, instead ensuring structure and sequence of information between the qualitative and quantitative components of the study.

The introduction requires restructuring to prioritize presenting essential information without unnecessary additions. Similarly, the results section should be organized to sequentially present findings from both qualitative and quantitative analyses. The discussion should be added and show the qualitative results and quantitative findings.

One more comment is to use the same concept “Nannies” or educators.

Thanks,

Ahmad Hanani
