# Supplementary material for: Clarifying terminology and definitions in education services for mental health users: A disambiguation study
Source: PLoS One. 2024 Jul 3;19(7):e0306539. doi: 10.1371/journal.pone.0306539 (PMC11221696; doi:10.1371/journal.pone.0306539)
Supplement: Attachment — Submitted filename: Response to Reviewers.docx [file pone.0306539.s005.docx]

Editor’s comments:

After a thorough examination of your manuscript, it is apparent that your work exhibits significant potential. I kindly request some minor revisions to further enhance the manuscript. These suggestions are tailored to pinpoint specific areas where refinement could enhance overall clarity and amplify the impact of your work. The detailed suggestions are provided below, and I kindly ask you to respond to both the editor and the reviewers. Please address each suggestion individually in a separate file and highlight the modifications made in the manuscript. Your attention to these revisions is greatly appreciated.

Thank you for allowing us to review the paper for publication in the journal. We have carefully considered all the suggestions, and believe they were beneficial for crafting an enhanced version of the manuscript, which is now more succinct and clear, with examples and connections.

To facilitate tracking changes in the response to reviewers, we have used blue colour coding and indicated the corresponding pages and lines within the document -manuscript with track changes-.

**Abstract**

The abstract, while informative, can be improved for clarity and conciseness. Here are some specific suggestions:

1. **Clearer Structure:** Begin with a concise statement on the issue of terminological comparability and the need for standardized classifications in service research. Follow with a brief explanation of the study's focus on education services and its alignment with the PECUNIA project.
2. **Streamlined Methodology Explanation:** Condense the explanation of the ontoterminology approach, focusing on the key steps without overwhelming detail. Emphasize the importance of accurate identification and disambiguation of services.
3. **Results Highlight:** Clearly state the key findings, such as the low percentage of accurate services after disambiguation. This will help readers quickly grasp the study's outcomes.
4. **Impact Statement:** Conclude with a concise statement on the significance of standardized methodologies and classifications in promoting comparability across sectors and ensuring a nuanced understanding of diverse services.
5. **Revised Keywords:** Ensure that the keywords reflect the main concepts of the abstract, such as disambiguation, education services, service research, and the DESDE system.

Thank you for the suggestion, the abstract has been revised according to indications to clearly and concisely inform readers about the study

In the wake of the mental health crisis in children and adolescents, the coordination of education and mental health services has become a global priority. However, differing terminologies and classifications across sectors, hinder effective comparison. The classification in education focuses mainly on outputs like qualifications or throughputs like teaching programs. This proof-of-concept study tested the applicability of a standard classification of health services, the Description and Evaluation of Services and DirectoriEs (DESDE), to evaluate education services for mental health users in the context of Spain and The Netherlands. It was conducted alongside the PECUNIA project, that sought to develop methods for the assessment of mental health costs and outcomes in different sectors. The study followed an ontoterminology approach involving: 1) identification of services from a predefined list of 46 resource-use items, 2) Disambiguation of identified services with the DESDE, and classifying them as accurate, ambiguous, vague or confuse; and 3) external validation by an expert panel. The analysis was conducted at the level of type of resource, target population and care provision. From the initial list, only ten of the resources could be categorized as services using DESDE, and not activities, interventions or professionals. Only four of them (8,65%) were accurate across all disambiguation categories. Experts were unaware of terminology problems in classification of service provision in the education sector. Classifications and glossaries can clarify service naming, description and costing allowing comparative effectiveness analysis and facilitating cross-sectoral planning. This should be grounded in common methodologies, tools, and units of analysis.

Keywords: Disambiguation, education services, service research, DESDE system, PECUNIA project **Introduction section**

The introduction provides a comprehensive overview of service science and its multidisciplinary nature, emphasizing the interconnectedness of various service sectors. Consider streamlining the introduction for better readability:

1. **Concise Background:** Condense the background information on service science and its multidisciplinary nature, focusing on its relevance to understanding complex interventions.
2. **Clarity on Systems Thinking:** Clearly articulate the concept of systems thinking and its application to service provision, giving examples without delving too deeply into specific empirical studies.
3. **Streamlined Structure:** Organize the introduction into shorter paragraphs, each addressing a specific aspect of service science, systems thinking, and the need for operational classifications. This will enhance overall clarity and engagement.
4. **Transition to the DESDE System:** Introduce the DESDE system and its role in providing a standardized classification for services, setting the stage for the study's focus on education services.

 Thank you for providing a very useful guide to organize the introduction. The whole section has been amended considering suggestions, and structured in different paragraphs separating service science, systems thinking and operational classifications. The first paragraph has been slightly modified to strengthen the importance of an integrated perspective of service provision. A new reference has been incorporated.

Pg3 lines 56-58. In the wake of the mental crisis in children and adolescents, the coordination of education and mental health services has become a global priority. Integrated youth services which rely on evidence-based models, are designed to provide effective care through a holistic approach (1). This integration is part of a broader discipline known as service science that has emerged in response to the growing complexity of the service sector, particularly in more developed economies (2).

1. Richards MC, Benson NM, Kozloff N, Franklin MS. Remodeling Broken Systems: Addressing the National Emergency in Child and Adolescent Mental Health. Psychiatric services. 2024 Mar 1;75(3):291–3. <https://doi.org/10.1176/appi.ps.20220283>

For the sake of clarity an example has been provided on how whole systems operate

Pg3 lines 68-72 ‘For example, improvements in health services may enhance educational outcomes by increasing student mental health, wellbeing etc. and conversely educational advancements can lead to better health awareness’.

Explanation of the DESDE system has been improved and moved to the end of the section, to facilitate the understanding of the classification and the connection with the objectives of the study.

Pg 6 lines 134- 143. ‘The Description and Evaluation of Services and DirectoriEs (DESDE) is an example of an international, intersectoral classification system valid for the evaluation of complex systems [19]. It originates from the field of mental health services research. In the past 25 years, it has been extended to incorporate other care sectors such as social [20], child and adolescent care, drug and alcohol, ageing, disabilities, long-term care [19,21]. DESDE has been validated and extensively used in the field of health services research and health economics for the comparison of resource utilization across different settings, efficiency analysis and the calculation of unit costs, among others [22]. Furthermore, DESDE is the only existing tool that provides local, bottom-up information that can be used across different sectors [5]’.

**The methods section**

It provides a comprehensive overview of the study's design, context, and application of the DESDE system. However, it can benefit from some improvements for clarity and conciseness:

1. **Alignment with PECUNIA Project Objectives:**

   Ensure a more explicit connection between the study's methodology and the broader objectives of the PECUNIA project. Clearly state how the study contributes to the overarching goals of the project, providing a seamless transition from project context to the study design.

The paragraph regarding the PECUNIA project has been reviewed to clarify the connection and synergy between both studies.

Pg7 lines 163-175 ‘The study was conducted alongside the ProgrammE in Costing, resource use measurement and outcome valuation for Use in multi-sectoral National and International health economic evaluAtions (PECUNIA) project (<https://www.pecunia-project.eu/>). PECUNIA was a European-funded consortium aimed at developing new standardised, harmonised and validated methods and tools for the assessment of costs and outcomes in European healthcare systems from a whole-system perspective including health and social care, employment, education and justice sectors. Although methods and tools developed within the PECUNIA project were meant to be generic, three mental health disorders (depression, post-traumatic stress disorder, and schizophrenia) were selected as illustrative examples. Building on the foundational goals of PECUNIA, this study’s focus on disambiguation and classification was related to the critical objectives of definition and harmonization within the project [11].The initial list of education resource-use items was compiled from literature reviews conducted within PECUNIA. Once classified with DESDE, these services were used in PECUNIA for cost calculation’.

2. **Axes in the DESDE System:**

   Offer a more comprehensive explanation of the axes in the DESDE system. While a multiaxial code structure is mentioned, briefly describe each axis to aid readers unfamiliar with DESDE, enhancing overall clarity and understanding.

A complete description of the axes has been included in the text

Pg 13 lines 286-296. ‘DESDE provides a multiaxial system that contains information on the following aspects of a service or a BSIC: a) the sector cluster (health, social, education, employment, justice); b) the target population, Including the age (e.g. CX – children and adolescents), gender, and the diagnosis group for which the service is intended. Code is based on the International Classification of Diseases 10^th^ edition (ICD-10) [37](e.g. F7-F8 - Mental retardation, disorders of psychological development), and the International Classification of Functioning, Disability and Health (ICF) [38]; c) a code of the main service function or type of care and its additional qualifiers. This code is the core component of DESDE (Fig 1). Two DESDE axes were used for the disambiguation study, target population and service type of care.’

3. **Expert Involvement Elaboration:**

   Elaborate on the specific contributions of each expert working group (A, B, C). Specify the unique perspectives or expertise each group brought to the study, providing a more nuanced understanding of expert involvement and its impact on the classification process.

The contributions of each expert group has been explained and incorporated into their corresponding subsection within the procedure.

Pg 14 lines 302-360:

‘Procedure

This ontoterminology study followed a multistep process to assess the clarity of terms in education services and involved three working groups:

Step 1 - Service identification

Group A comprised members from Maastricht University (IP, LJ, SE, AP, RD). They undertook peer-reviewed and grey literature searches, as well as multi-national expert surveys that took place in six European countries prior to this study, as part of the PECUNIA project [39]. (…)

Step 2 - Disambiguation

Group B included two experts in disambiguation using DESDE (MR, LS) that analysed the list compiled by group A. (…) The process involved two levels:

- 1. Defining the unit of analysis (type of resource): group B reviewed the general list and ensured that the items and their descriptions corresponded to the same unit of analysis (commensurability), in this case ‘services’ and not to other units of analysis such as interventions, professionals, or outcomes. (…)

2. DESDE classification: group B assessed information about the target population - including age and diagnosis group-, as well as the type of care provided in the services. (…)

Step 3- External validation

To finalize the disambiguation process and evaluate the suitability of DESDE for classifying education services, group C was consulted. This group consisted of a panel of external experts recruited from the current study’s co-authors’ professional network using purposive sampling (two from Spain, and three from the Netherlands). It included two academics, one public agent from the regional Ministry of Education and two third-sector professionals (AG, AR, TS, IO, MS). Their primarily role was to validate the services coded by group B, using their deep knowledge of the sector.

Consultation involved individual semi-structured interviews. Experts were guided through a series of generic inquiries about education within the contexts of Spain and The Netherlands. Interview questions were pre-defined by the co-authors of the current study. (S1 Table 1). Experts critically reviewed the comprehensiveness and accuracy of the service list and verified the correctness of the assigned codes, taking into account the educational realities in both countries.

4. **Qualitative Data Integration:**

   If applicable, consider integrating qualitative data or quotes from expert consultations to enrich the narrative. This could offer a more vivid portrayal of the challenges faced and solutions proposed during the expert consultation process, enhancing the depth of the methodological description.

This information has been included as part of the results of the study

5. **Contextual Clarification:**

   Provide a brief, clear explanation of the PECUNIA project and its objectives before delving into the study design. This will help readers understand the broader context of the research and appreciate the study's relevance to the project's overarching goals.

This suggestion has been integrated in response to point 1.

6. **Conciseness in Study Design:**

   Streamline the description of the study design, emphasizing its alignment with the PECUNIA project. Highlight specific mental health disorders chosen for illustration without unnecessary detail, ensuring a balance between clarity and conciseness.

The section addressing the study design has been deleted, the information previously included in this section has been move to the beginning of the method.

Pg8 lines157-162 ‘The study was designed as a proof-of-concept to test the applicability of the DESDE classification system to the education sector. It aimed to assess the clarity of service terms in this sector using an ontoterminology approach. Meaning the study of disambiguation of technical and scientific terms using standard classifications [24]. This method helps identify precise definitions and relationships between terms, enhancing the retrieval of domain-specific information. It also aids in creating controlled vocabularies, classifications, and glossaries [24]’.

7. **Clarity in DESDE Application:**

   Clearly state the rationale for applying the DESDE classification to education services and explain how the coding process was conducted using a generic list of prototype services. Ensure that the application of DESDE is presented with clarity and relevance to the study.

Rationale for the application of the DESDE system has been included in the study design.

Pg 14, lines 299-303. ‘In this proof of concept study, the rationale for applying DESDE classification arose from the need to create a standardized framework for describing and assessing education services in a comparable manner. DESDE was originally developed for use in health and social care sectors. It’s methodological rigor made it ideal to bring similar benefits of standardization to the education sector’.

8. **Simplified Education Contexts:**

   Condense detailed descriptions of education contexts in Spain and The Netherlands to focus on key aspects relevant to the study. Make the information more accessible to readers without overwhelming them with unnecessary details.

The structure of educational contexts in Spain and in The Netherlands have been refined and reduced.

Pg9 lines 195-223 In Spain, education is mandatory from 6 to 15 years [25]. Pre-school education while not compulsory, is available for children from birth to 6 years, and nursery school (0-3 years) and infant school (3 to 6 years)[25]. OECD reports indicate that in Spain 95% of 3 years old and 97% of 4 years old are enrolled in pre-school [26]. Education continues with primary school from ages 6 to 12, followed by secondary education until age 15. Post-secondary options such as upper secondary education or vocational training are available for 16 to 18 years, but are not compulsory [25]. Special education is organized in three sections: special early childhood education, adapted compulsory basic education, and training programs for transition to adult life from 16 to 20 years old, and it is not compulsory [27].

In the Netherlands, school attendance is compulsory from 5 to 16 years old, and for those between 16 and 18 who have not yet met basic qualifications [14]. Pre-school education is not compulsory [28]. Children typically start primary school at the age of 4 and transition to secondary education at 11-12. Secondary school education is structured into three distinct tracks [28]: pre-vocational education lasts 4 years and prepares students for entering vocational schools [29]; Higher general continued education takes 5 years and prepares students to enter a university of applied sciences; preparatory scientific education lasts 6 years and prepares students to enter an academic university [30]. Special education is delivered in separate special schools and is organized into four clusters to address specific needs: visual impairments, hearing or speech impairments, physical or mental handicaps , and mental disorders and behavioural problems [31].

9. **Streamlined Governance Structure:**

   Streamline governance structure information for both countries, emphasizing key points relevant to education services. Ensure that the governance structure is presented in a clear and concise manner.

The text regarding the governance structure in both countries has been reviewed and shortened to highlight the most important information.

Pg 11-12 lines 225-250. In Spain, the Ministry of Education, Culture and Sport governs the education sector. Individual schools and budget are managed by the autonomous communities with guidelines set by the central government. Public schools receive government subsidies and must adhere closely to Ministry requirements. Private schools which may be subsidised or not, also depend on and are authorised by the regional government, but they have their own management. Although Spanish universities operate independently from the government, they are regulated and monitored by the national and regional quality and accreditation agencies [26].

In the Netherlands, the Ministry of Education, Culture and Science, governs the education sector, setting general rules while allowing institutes freedom in their teaching methods [32]. Primary and secondary schools are further governed by school boards, with several schools falling under one school board. The quality of education is monitored nationally by the Inspectorate of Education, and funding for schools is allocated based on the number of students; with additional support for disadvantaged students or students with special needs [33]. Both public and private schools can receive government funding if they meet statutory requirements. Vocational and higher education institutes operate as separate legal entities and receive funding through a combination of tuition fees and government grants, which are based on the institution’s performance. Tertiary educational institutions are governed by boards that formulate and implement strategies within the government’s framework [33].

10. **Professional Qualifications Overview:**

    Briefly outline the qualifications and roles of key professionals in the education sector in Spain and The Netherlands. Ensure clarity and relevance to the study without delving into unnecessary details.

The qualifications and roles of key educational professionals have been succinctly outlined with irrelevant details removed to enhance clarity.

Pg 12-13, lines 252-279. ‘In Spain, the main reference professional in public schools is the teacher/educator. They must complete four years of university studies, and pass an official exam. Experience and training provide points that allow them to move between institutions. Special education adopts, the multitrack model described by Nash and Norwich [34], where all teachers receive generic training followed by specialized training therapeutic pedagogy or hearing and language [35]. In the private sector, a master’s degree is required in secondary though the official exam is not.

In the Netherlands, primary school teachers need a 4-year degree from either a university of applied sciences or a regular university. Secondary school teachers, are differentiated by the level of education they can teach, depending on whether they have a first degree (lower education levels e.g pre-vocational education) or second degree (advanced educational levels e.g. preparatory scientific education). Teaching assistants, that support the delivery of education at primary and secondary schools, must complete a vocational education degree [36]. In special education, although the level of education might be lower compared to regular education, the pedagogical requirements are more stringent. Higher education professionals are often required to have a degree from a university of applied sciences or a regular university, along with a university teaching qualification, though there are no specific statutory requirements. [36].

11. **DESDE System Explanation:**

    Provide a succinct yet comprehensive explanation of the DESDE system, its axes, and how it was utilized in the study. Include a concise description of the multiaxial code structure for a clearer understanding.

Please refer to point 2.

12. **Procedure Overview Streamlining:**

    Streamline the description of the assessment procedure, emphasizing key steps involved in service identification, disambiguation, and code development. Ensure clarity and simplicity in presenting the procedural overview.

13. **Expert Involvement Clarification:**

    Clearly outline the roles of the three working groups (A, B, C), providing a concise overview of their contributions to the study. Avoid redundancy and ensure that each group's role is distinctly communicated.

14. **Step-by-Step Process Breakdown:**

Break down the multistep process into clear, sequential steps, each with a succinct description. Ensure clarity in terminology, such as the distinction between disambiguation and development of prototype codes, for a more accessible presentation.

 Procedure has been modified to emphasise key steps, to clearly outline the roles of the groups involved, and incorporate indications provided in points 12-14. A new reference has been included.

Pg 14-17, lines 308-376 ‘ This ontoterminology study followed a multistep process to assess the clarity of terms in education services and involved three working groups:

Step 1 - Service identification

Group A comprised members from Maastricht University (IP, LJ, SE, AP, RD). They undertook peer-reviewed and grey literature searches, as well as multi-national expert surveys that took place in six European countries prior to this study, as part of the PECUNIA project [39]. The aim was to build a list of resource-use items in the education sector along with their respective definitions relevant for mental health economic evaluations [39]. These identified services in the education sector, along with services in other sectors, [40], formed the basis for developing new costing instruments such as the Resource Use Measurement (RUM) instrument, Reference Unit Cost (RUC) Templates, and RUC Compendium. [41-43].

Step 2 - Disambiguation

Group B included two experts in disambiguation using DESDE (MR, LS) that analysed the list compiled by group A. In terminology, disambiguation is the process of making something clear and resolving ambiguity and vagueness of terms [44]. Ambiguity refers to the terms that can be interpreted in more than one way because of imprecise definitions. Vagueness refers to the terms that are not sufficiently specified and therefore cannot be translated into a code. Our team established a threshold for differentiating ambiguity and vagueness in the analysis of health services using DESDE [44,45]. A service that could be assigned two codes of the reference classification system was considered ambiguous, whilst a service that could be assigned more than two codes, was categorized as vague. Confusion in this study refers to the terms whose definition did not allow classification. Items were categorized into ‘accurate’, ‘ambiguous’, ‘vague’, or ‘confusing’ based on descriptions. The process involved two levels:

- 1. Defining the unit of analysis (type of resource): group B reviewed the general list and ensured that the items and their descriptions corresponded to the same unit of analysis (commensurability), in this case ‘services’ and not to other units of analysis such as interventions, professionals, or outcomes. The standard description of services provided in DESDE was used to ensure that items could be compared like-with-like. Items classified as non-services were excluded from further disambiguation.

2. DESDE classification: group B assessed information about the target population - including age and diagnosis group-, as well as the type of care provided in the services. This enabled the assignment of prototype DESDE codes and additional qualifiers.

Step 3- External validation

To finalize the disambiguation process and evaluate the suitability of DESDE for classifying education services, group C was consulted. This group consisted of a panel of external experts recruited from the current study’s co-authors’ professional network using purposive sampling (two from Spain, and three from the Netherlands). It included two academics, one public agent from the regional Ministry of Education and two third-sector professionals (AG, AR, TS, IO, MS). Their primarily role was to validate the services coded by group B, using their deep knowledge of the sector.

Consultation involved individual semi-structured interviews. Experts were guided through a series of generic inquiries about education within the contexts of Spain and The Netherlands. Interview questions were pre-defined by the co-authors of the current study (S1 Table1). They critically reviewed the comprehensiveness and accuracy of the service list and verified the correctness of the assigned codes, taking into account the educational realities in both countries.

Reference

46. Lukersmith S, Taylor J, Salvador-Carulla L. Vagueness and ambiguity in communication of case management: A content analysis in the Australian national disability insurance scheme. International journal of integrated care. 2021 Mar; 21(1). <https://doi.org/10.5334/ijic.5590> PMID: 33776606

**Results section**

1. **Lack of Clarity in Disambiguation Criteria:**

The criteria for disambiguating services appear somewhat subjective and may benefit from a more explicit and standardized approach. Providing clearer guidelines for categorizing items as accurate, ambiguous, vague, or confusing could enhance the transparency and reproducibility of the classification process. Clearly articulate the criteria for classifying items into accurate, ambiguous, vague, or confusing categories. Consider providing illustrative examples to guide readers and potential future researchers in understanding the disambiguation process.

The definition of ‘ambiguity’ for classifying items, was established by a consensus group within the POMONA project, which focused on developing and rating health indicators for intellectual disability. This term has been incorporated into published papers. This collaborative effort provided a foundation for disambiguation. Additionally, our research group has developed and applied a framework for differentiating between ambiguity and vagueness in health services. The text has been modified to clarify this aspect.

chrome-extension://efaidnbmnnnibpcajpcglclefindmkaj/https://webgate.ec.europa.eu/chafea_pdb/assets/files/pdb/2004130/2004130_1_en.pdf

Pg 16 lines 338-343. ‘Our team established a threshold for differentiating ambiguity and vagueness in the analysis of health services using DESDE (26,46). A service that could be assigned two codes of the reference classification system was considered ambiguous, whilst a service that could be assigned more than two codes, was categorized as vague. Confusion in this study refers to the terms whose definition did not allow classification’.

2. **Limited Exploration of Ambiguous and Vague Categories:**

The Results Section could delve deeper into the ambiguous and vague categories, providing more detailed examples and insights into why certain items fell into these classifications. A more in-depth analysis of these categories could shed light on the intricacies of classifying educational services. Dedicate more space to discussing items categorized as ambiguous or vague. Offer nuanced explanations for why certain items fell into these categories, providing contextual details that highlight the challenges faced during the classification process.

The disambiguation section within the results has been expanded and clarified to better address the categories used for disambiguation

Pg 17 lines 387-405

Step 2 –Disambiguation

Level 1 - Unit of analysis (commensurability)

In the first step of disambiguation, ten items (22%) were classified as accurate and included in the subsequent disambiguation steps. Thirty-five items (76%) were classified as confusing, because they did not refer to services but rather to procedures (‘special needs diagnostics’), professionals (‘liaison teacher’) or consequences (‘low school adaptation/competence’) and were therefore excluded from the subsequent disambiguation steps. One item (2%), ‘student counselling’, was excluded because it was not clear what was understood by counselling in this term and admitted very different units of analysis. Please refer to S1 Table 2 for more details.

Level 2 - DESDE classification

Ten items, initially classified as accurate in terms of unit of analysis and thus defined as “services” were further assessed for clarity concerning the target population. Five items were classified as accurate (50%). Two items were classified as ambiguous (20%) these included ‘special education services’ and ‘student transport to special education facility’ where both primary and secondary students could be admitted. Another two items were classified as vague (20%) because they lacked specific descriptions of age or diagnosis groups (‘special education day school’ and ‘day school’). One item was classified as confusing (10%) due to an incoherent description of the target population; ‘higher education school (university, college, vocational school)’ could include not only adults, but also adolescents. Please refer to Table 1 for more details.

Regarding service type, eight items were classified as accurate (80%). One item, ‘education support in a private setting’ was classified as ambiguous (10%) due to it having two different codes, with the second code varying by the frequency of care provided. Another item, ‘special education services’ was classified as vague (10%), because it required a wide range of different codes. For more details, please refer to Tables 1 and 2.

3. **Insufficient Exploration of Expert Suggestions:**

While expert opinions are briefly mentioned, the Results Section could benefit from a more comprehensive exploration of how expert feedback influenced the classification process. Explicitly linking expert comments to specific items in the disambiguation process would provide a clearer understanding of the impact of expert validation. Integrate expert comments more seamlessly into the narrative. Explicitly connect expert suggestions to specific items in the disambiguation process, emphasizing the role of expert validation in refining the classification criteria.

The paper includes information on expert validation in table 2, and an additional section has been added to address the main results. In the discussion, a comprehensive paragraph has been included, detailing expert suggestions and their impact on the classification process.

Pg 22-23 lines 422-472 ‘Initially, the five experts from the panels (group C) were unaware of any terminology problem in the classification of educational services. Only one expert from Spain highlighted potential problems when comparing services between regions in the country. Experts considered ‘special education services’ and ‘higher education school’ umbrella terms that could refer to a variety of settings and discarded them as they were too broad to be useful for an operational classification. Regarding special education, they informed that in recent years there has been a shift towards a more inclusive school environment both in Spain and The Netherlands. Students with special education needs are educated in regular schools with additional support if required. For example, in the Netherlands regular schools receive additional funds to support students with special needs. Furthermore, partnerships between regular and special education schools facilitate the integration of well-performing students into regular schools. In Spain there were special centres for students with autism spectrum disorders and intellectual disabilities among other conditions, but the trend in the last decades has been to offer universal education in regular schools with specific programmes for the students with special needs. Only individuals with more severe disabilities are typically accommodated in ‘special education day schools’. Experts indicated that this distinction should be reflected in the DESDE code axis of the target population. Therefore, the ICF severity code (ICF3), was added in this case. ‘Boarding school’, ‘special education boarding school’, while no longer prevalent in public systems, were considered accurate int their definition and code. Similarly, ‘day school’ and ‘Student transport to special education facility’ were considered appropriate’. (…)

4. **Inconsistent Terminology:**

The use of terms like "accurate," "ambiguous," "vague," and "confusing" could be standardized throughout the section for better coherence. Ensuring consistent language will improve the overall readability and interpretation of the results. Ensure consistent use of terminology throughout the Results Section. Streamline the language used to describe the classification outcomes, making it easier for readers to follow and interpret the findings.

Definitions of the categories -accurate, ambiguous, vague and confusing has been included in the method section. The results section has been revised to ensure clear understanding and application of these categories.

**The Discussion Section**

1. **Limited Exploration of Expert Suggestions:**

While the discussion briefly touches upon expert suggestions, it could benefit from a more detailed examination of how expert consultations influenced the refinement of service descriptions and the application of DESDE codes. Explicitly linking expert feedback to specific challenges or improvements in the classification system would enhance the discussion. Dedicate a section to thoroughly explore the impact of expert consultations on the classification system. Provide specific examples of services that were refined based on expert input and discuss how this collaboration improved the accuracy of the classification.

The section has been modified to explicitly link experts feedback and results. Please note that the experts validated the assigned codes, but the suggested modifications to the instrument have not been implemented in this study.

Pg 28 lines 509-520

Discussion: Expert consultation was in this sense crucial for nuanced service descriptions, appropriate code assignments, and gaining deeper insights into the national education systems from a bottom-up perspective. Their input, based on their contextual background, contributed significantly to refining the outputs of the study and enhance the reliability of the findings: : ‘boarding school’ and ‘special education boarding school’, were considered correctly classified despite being residual and absent from the public sector of both countries. Additionally, ‘day school’ and ‘student transport to special education facility’ were accurate at the level of DESDE code, although the latter lacked specificity regarding target population due to unspecified age groups. ‘Education support at your place of study’, and ‘education support in a private setting’ received high intensity codes within the provided range. Finally, ‘special education day school’ required a code for severity. ‘Higher education’ and ‘special education’, were considered umbrella terms and not useful for classification.

Suggestions made by experts for adapting the DESDE system to the education sector included the future development of new codes to differentiate day and evening school, reflecting differences in operational contexts such as hours, staffing, or resources. As well as differentiating regular education from special services provided within regular school premises. However, the information obtained in the study is established as the first step for the development of a classification of education services that will enable comprehensive mapping of the education sector in specific geographical areas’.

2. **Insufficient Exploration of Service Accuracy Levels:**

The discussion provides the overall percentage of services accurate at different levels of disambiguation, but a deeper analysis of the implications and challenges associated with these accuracy levels is warranted. Discussing specific examples or patterns in services that were accurate or faced challenges at different levels would provide a richer understanding. Conduct a more nuanced analysis of the accuracy levels reported, discussing specific services that excelled or faced challenges at different stages of disambiguation. This would provide a more comprehensive understanding of the classification process.

Thank you for the comment. The inclusion of a paragraph explaining the implications of accurate classification of services enhances the depth and relevance of the discussion. It provides a broader perspective on the implications of the study’s findings.

Pg27 lines 487-497 ‘The magnitude of the terminological problem in the education sector is at least as relevant as it is in the health and social sectors. From the original list of 46 resource-use items only ten were typified as actual services (resource inputs) while other terms referred to professionals, interventions (throughputs), and consequences (outputs). Five services were accurate at target population level and eight were accurate at the level of service type (DESDE code). The identification of only four services as accurate at all levels of disambiguation (8,65%) namely ‘boarding school’, ‘special education boarding school’, ‘education support at your place of study’ and ‘night school’, highlights the prevalence of ambiguity within the education sector. This ambiguity reflects the complexity and diversity of education services and the challenges inherent in defining and categorizing them. Accurate classification informs policy development by providing policymakers with a clear understanding of the types of services available and the population they serve. This allows for a more effective resource allocation and collaboration across sectors’.

3. **Limited Exploration of Code Adaptation Suggestions:**

The discussion briefly mentions suggestions for adapting the DESDE system to the education sector, such as the need for new codes to differentiate day and evening school. Further exploration and discussion of these suggested adaptations, including potential implications and challenges, would enhance the completeness of the discussion. Elaborate on the suggestions for adapting the DESDE system to the education sector. Discuss the rationale behind proposed changes, potential benefits, and challenges associated with incorporating new codes or modifying existing ones.

The paragraph on the future additions/modifications of the DESDE tool have been mentioned but please note that this study represents the first step where the main point was validating the assessed services and test the applicability of the current system to the education sector and not a full development of the classification system.

Pg 29 lines 521-529. Suggestions made by experts for adapting the DESDE system to the education sector included the future development of new codes to differentiate day and evening school, reflecting differences in operational contexts such as hours, staffing, or resources. As well as differentiating regular education from special services provided within regular school premises. However, the information obtained in the study is established as the first step for the development of a classification of education services that will enable comprehensive mapping of the education sector in specific geographical areas. This standardized framework will facilitate systematic data collection and better comparison on the availability of services in a region at every level of the ecological system (micro, meso and macro)’.

4. **Incomplete Integration of ISCED System:**

The discussion mentions the potential complementarity of international classifications like DESDE and ISCED but does not delve into the practical implications of integrating these systems. Further exploration of how combining information from DESDE and ISCED could enhance the understanding of the education sector would strengthen the discussion. Explore in detail how integrating information from the DESDE and ISCED systems can provide a more holistic view of the education sector. Discuss specific examples where this combination could enhance the classification and understanding of educational services.

A short paragraph has been included regarding the benefits of combining different international classifications, like DESDE and ISCED. An example is also provided for clarification (a new reference has been added):

Pg29 lines 531-544. ‘Integrating international classifications has demonstrated a potential for disambiguation and therefore improving the comparability of resources across settings [20,44]. The combined use of complementary classification systems of services, interventions and professionals has proved its value in health (e.g. psychotherapy) [44] and social care (e.g. case management) [45]. In education, the description of service provision using DESDE can be combined with information on programs and qualification levels from ISCED and the types of professionals involved (ISCO-International Standard Classification of Occupations [47]. Thus, a more comprehensive overview of the education sector could be achieved in evaluating a primary school for special education, DESDE could provide insights into the facilities structured day care (i.e D4.2 day care for a fixed number of hours/days); ISCED could clarify the level of education offered such as primary education focusing on basic skills like reading, writing and mathematics (ISCED level 1); and ISCO could detail the roles of professionals, like special education teachers (ISCO code 2340). This approach would enhance comparability and support evidence-informed decision-making [13]’

47. International Standard Classification of Occupations. ISCO-08 Structure, Index Correspondence with ISCO-88. ISCO; 2008. Available: <https://www.ilo.org/publications/international-standard-classification-occupations-2008-isco-08-structure>

5. **Practical Implications:**

Expand on the practical implications of the study's findings for policymakers, educators, or researchers. Discuss how the standardized classification system could contribute to more effective educational service planning and delivery.

 A paragraph has been added, highlighting benefits for the education sector and professionals involved, and the potential connection with other sectors.

Pg 29 lines 569-576. ‘As the first step, this proof of concept is important to set the ground for conducting a comprehensive mapping of an area and to study the feasibility and expanding the classification to encompass the entire education system. Standardized data enables policymakers and educators to make decisions based on robust evidence thereby enhancing effective service planning and delivery. This. Additionally, adopting a standardized classification for a whole system approach, would facilitate deeper integration with other sectors like healthcare, ensuring that policies and practices are interconnected across sectors, enhancing evidence-informed decision-making and optimizing allocation of resources’.

Reviewers' comments:

Reviewer's Responses to Questions

**Comments to the Author**

1. Is the manuscript technically sound, and do the data support the conclusions?

The manuscript must describe a technically sound piece of scientific research with data that supports the conclusions. Experiments must have been conducted rigorously, with appropriate controls, replication, and sample sizes. The conclusions must be drawn appropriately based on the data presented.

Reviewer #1: Yes

Reviewer #2: Partly

2. Has the statistical analysis been performed appropriately and rigorously?

Reviewer #1: N/A

Reviewer #2: Yes

3. Have the authors made all data underlying the findings in their manuscript fully available?

The [PLOS Data policy](https://urldefense.com/v3/__http:/www.plosone.org/static/policies.action*sharing__;Iw!!D9dNQwwGXtA!RSmsLEhUWdGoM1vyHu2W3ivQhrP00LSw63FJ1ifbarpZzIf3BD9TjapezpN3mcUKhmCeoQt4w9xyffQAso0M$) requires authors to make all data underlying the findings described in their manuscript fully available without restriction, with rare exception (please refer to the Data Availability Statement in the manuscript PDF file). The data should be provided as part of the manuscript or its supporting information, or deposited to a public repository. For example, in addition to summary statistics, the data points behind means, medians and variance measures should be available. If there are restrictions on publicly sharing data—e.g. participant privacy or use of data from a third party—those must be specified.

Reviewer #1: Yes

Reviewer #2: Yes

4. Is the manuscript presented in an intelligible fashion and written in standard English?

PLOS ONE does not copyedit accepted manuscripts, so the language in submitted articles must be clear, correct, and unambiguous. Any typographical or grammatical errors should be corrected at revision, so please note any specific errors here.

Reviewer #1: Yes

Reviewer #2: Yes

5. Review Comments to the Author

Please use the space provided to explain your answers to the questions above. You may also include additional comments for the author, including concerns about dual publication, research ethics, or publication ethics. (Please upload your review as an attachment if it exceeds 20,000 characters)

Reviewer #1: This is a highly worth while study establishing a proof of concept in relation to the use of robust classification and terminology (taxonomical framework) to the area of school/education services for mental health service users with broader implications for systems analysis in education.
The authors have used existing an evidence based tools with expert analysis to assess the terminology used in education services and found considerable ambiguity and confusion. The outcomes of this work lay a basis for the development of standardised classification systems for comparison, planning and decision making in a key area of human services, namely education.

One minor point, the authors refer to three levels in the proc less of disambiguation - but there appeared to be four in the tables and discussion - namely accurate, vague, confusing and ambiguous. This needs to be clearer in the outline of "The Procedure" Step 2.

Thank you for your comments. The paragraph has been modified to make it clearer.

Pg16 line 345 ‘Items were categorized into ‘accurate’, ‘ambiguous’, ‘vague’, or ‘confusing’ based on descriptions. The process involved two levels:’

Reviewer #2: Dear Editor,

I hope this letter finds you well.

I would like to express my gratefulness for your trust and for giving me the opportunity to review the manuscript titled "Clarifying Terminology and Definitions in Education. no. PONE-D-23-36992 ".
According to objectives of the study "to test the applicability of a standard classification", and the Description and Evaluation of Services and DirectoriEs (DESDE), to evaluate education services for mental health users in the context of Spain and The Netherlands.
The title holds significant importance, as do the objectives of the study. However, here are some comments about where enhancements can be made to the manuscript.
1- Method need to clarify and demonstrate the structural formula employed in comparing the educational services between the two countries under examination. This clarification will provide a clearer understanding of the methodology employed and facilitate a more comprehensive analysis of our findings.

The method regarding expert validation has been extended and clarified. Please note that while experts provided a context in both Spain and The Netherlands to inform their evaluation, this background served mainly as a reference point for assessing the accuracy of the service list and the assignment of codes. We did not undertake a detailed mapping of education services in these two countries.

Pg 16 lines 359-376

‘Step 3. Expert validation

To finalize the disambiguation process and evaluate the suitability of DESDE for classifying education services, group C was consulted. This group consisted of a panel of external experts recruited from the current study’s co-authors’ professional network using purposive sampling (two from Spain, and three from the Netherlands). It included two academics, one public agent from the regional Ministry of Education and two third-sector professionals (AG, AR, TS, IO, MS). Their primarily role was to validate the services coded by group B, using their deep knowledge of the sector.

Consultation involved individual semi-structured interviews. Experts were guided through a series of generic inquiries about education within the contexts of Spain and The Netherlands. Interview questions were pre-defined by the co-authors of the current study (S1 Table 1). They critically reviewed the comprehensiveness and accuracy of the service list and verified the correctness of the assigned codes, taking into account the educational realities in both countries.’

2- In the discussion part need to provide a detailed comparison between Spain and The Netherlands. This comparative analysis will light on any differences and contribute to a deeper understanding of the study’s outcomes.

Thank you for your comment. It is important to emphasize that the primary goal of the study, was not to compare services across different areas but rather to validate the classification system’s suitability for categorizing services identified through literature searches. The input from experts, based on their contextual backgrounds, was instrumental in validating the provided codes and ensuring their applicability to the identified services. The contribution of the study is to establish the basis for the development of a classification framework within the education sector. Considering this aspect the discussion has been refined.

Pg 28 lines 510-520

‘Expert consultation was in this sense crucial for nuanced service descriptions, appropriate code assignments, and gaining deeper insights into the national education systems from a bottom-up perspective. Their input, based on their contextual background, contributed significantly to refining the outputs of the study and enhance the reliability of the findings: ‘boarding school’ and ‘special education boarding school’, were considered correctly classified despite being residual and absent from the public sector of both countries. Additionally, ‘day school’ and ‘student transport to special education facility’ were accurate at the level of DESDE code, although the latter lacked specificity regarding target population due to unspecified age groups. ‘Education support at your place of study’, and ‘education support in a private setting’ received high intensity codes within the provided range. Finally, ‘special education day school’ required a code for severity. ‘Higher education’ and ‘special education’, were considered umbrella terms and not useful for classification.

Suggestions made by experts for adapting the DESDE system to the education sector included the future development of new codes to differentiate day and evening school, reflecting differences in operational contexts such as hours, staffing, or resources. As well as differentiating regular education from special services provided within regular school premises. However, the information obtained in the study is established as the first step for the development of a classification of education services that will enable comprehensive mapping of the education sector in specific geographical areas’.

Thank you once again for your consideration.
Sincerely,
Ahmad Hanani

6. PLOS authors have the option to publish the peer review history of their article ([what does this mean?](https://urldefense.com/v3/__https:/journals.plos.org/plosone/s/editorial-and-peer-review-process*loc-peer-review-history__;Iw!!D9dNQwwGXtA!RSmsLEhUWdGoM1vyHu2W3ivQhrP00LSw63FJ1ifbarpZzIf3BD9TjapezpN3mcUKhmCeoQt4w9xyffxGdgqt$)). If published, this will include your full peer review and any attached files.


If you choose “no”, your identity will remain anonymous but your review may still be made public.


**Do you want your identity to be public for this peer review?** For information about this choice, including consent withdrawal, please see our [Privacy Policy](https://urldefense.com/v3/__https:/www.plos.org/privacy-policy__;!!D9dNQwwGXtA!RSmsLEhUWdGoM1vyHu2W3ivQhrP00LSw63FJ1ifbarpZzIf3BD9TjapezpN3mcUKhmCeoQt4w9xyfezRgEDB$).

Reviewer #1: No

Reviewer #2: No
